# Round Table Discussion: Aesthetic Treatment Considerations for the Perimenopausal & Menopausal Patient

**DOI:** 10.1111/jocd.70626

**Published:** 2025-12-29

**Authors:** Sabrina G. Fabi, Maya Firsowicz, Payvand Kamrani, Zoe D. Draelos, Steven H. Dayan

**Affiliations:** ^1^ University of California San Diego Medical Center San Diego California USA; ^2^ Dermatology Institute Chula Vista California USA; ^3^ Aesthetic Solutions Chapel Hill North Carolina USA; ^4^ Dermatology Consulting Services, PLLC Duke University High Point North Carolina USA; ^5^ University of Illinois Chicago Illinois USA

**Keywords:** estrogen, estrogen‐deficient skin, menopause, perimenopause, roundtable discussion, therapeutic options

## Abstract

**Background:**

Menopause is a normal feature of the female aging process that commonly occurs during the 6th decade of life. During perimenopause, many negative effects begin to occur affecting the skin that are associated with decreasing production of estrogen, progesterone, and testosterone. Estrogen supports the extracellular matrix, collagen production, and dermal blood supply, while progesterone promotes extracellular matrix formation, dermal blood supply, and dermal water retention. Decreased testosterone impacts cutaneous blood supply and sebum production, causing skin dryness.

**Aims:**

The primary objective of the following consensus roundtable was to discuss best practices for aesthetic providers when treating patients with perimenopausal and menopause‐related skin conditions.

**Patients/Methods:**

A roundtable discussion was held by several notable experts in their field during a special edition of the *Thriving in Diversity* webinar series on Friday, June 13, 2025. The discussion included a gynecologist, a plastic surgeon, and two dermatologists who provided their clinical experience and consensus recommendations for treating perimenopausal and menopausal patients.

**Results:**

Numerous therapies are available to treat patients with perimenopausal and menopause‐related skin conditions. These include hormone replacement therapy (HRT), topical estrogen therapy, topical retinoids, topical cosmeceuticals (moisturizers, signal peptides, growth factors, exosomes), dermal injectables and biostimulators, and energy‐based treatments, each with varying beneficial effects. The experts in this panel find that hormone replacement therapy, topical estrogen, injectable poly‐L‐lactic acid, injectable hyaluronic acid, and carbon dioxide laser resurfacing are the most effective interventions for treating hormone‐related skin changes.

**Conclusions:**

Although menopause ultimately affects all women and is likely to produce unwanted effects including undesirable aesthetic changes, numerous treatments are available to treat or mitigate these effects.

## 1 Introduction

Menopause is a normal stage of female aging that commonly occurs during the 6th decade of life [1, 2]. It is defined as the complete cessation of menstrual periods for 12 months and is associated with characteristic systemic symptoms including hot flashes, night sweats, insomnia, and mood changes [1]. The transition period leading to menopause, known as perimenopause, involves similar symptoms and can last 4 to 8 years. Compared with premenopausal women, the symptoms brought on by menopause and perimenopause are associated with significant decreases in mental and physical health‐related quality of life [3, 4]. Increasingly discussed are the negative effects menopause has on the skin.

Menopausal and peri‐menopausal symptoms are due to the decreased ovarian secretion of estrogens, androgens, and progesterone [5]. Prior to menopause, these hormones play a central role in skin health and wellness. Estrogen is well known to support the extracellular matrix, collagen production, and blood supply [6]. Progesterone likewise promotes formation of the extracellular matrix, blood supply to the skin, and dermal water retention [7]. When these hormone levels begin to decline during onset of perimenopause, skin changes become notable. Decreasing estrogen levels correlate with a decline in collagen [8]. Collagen levels in women decrease by about 1% per year beginning in early adulthood [9], becoming significant by the 5th decade [10]. Women lose approximately 30% of the collagen in their skin during the first 5 years of menopause and about 2% of collagen every year thereafter [11], impacting skin thickness and ultimately facial attractiveness [12].

Perimenopause and menopause are also associated with diminishing levels of progesterone. While this is responsible for some of the classic symptoms of menopause such as feeling more irritable and having sleep disturbances, it also impacts the skin by affecting sebum production and contributing to skin dryness [7]. There is also a gradual loss of testosterone during menopause that can cause dysphoria, unexplained fatigue, change in sexual function including reduced libido, changes in cognition, vasomotor symptoms, bone loss, and decreased muscle strength [13]. For the skin, a decrease in testosterone likewise can impact cutaneous blood supply and sebum production, negatively impacting both the perfusion and dryness of the skin [14].

These hormone‐driven changes represent macroscopic processes that visibly alter skin structure and function. Beneath the surface, aging is further orchestrated by fundamental molecular mechanisms that impair cellular integrity and resilience. Collectively referred to as the 12 “hallmarks of aging,” these include processes such as genomic instability, telomere attrition, epigenetic alterations, and mitochondrial dysfunction [15]. These hallmarks are organized into three categories: primary hallmarks, which trigger the initial molecular damage; antagonistic hallmarks, which attempt to compensate for damage but can become harmful when dysregulated; and integrative hallmarks, which represent the ultimate culprits of tissue dysfunction and organismal aging [15]. Together, these molecular events, operating downstream of hormonal shifts, and alongside other factors such as genetics and ultraviolet exposure [16], contribute to the progressive decline in skin health observed during menopause.

Altogether, treating the symptoms of hormone‐deficient skin can improve skin health and quality of life for both perimenopausal and menopausal women [6]. The objective of this article is to describe changes in skin quality and appearance associated with perimenopause and menopause, and to identify therapeutic options and their proposed mechanisms for treating or mitigating menopause‐related skin changes.

## 2 Materials and Methods

A roundtable discussion was held by four notable experts in their field during a special edition of the *Thriving in Diversity* webinar series on Friday, June 13, 2025 [17]. Two expert dermatologists, a gynecologist, and a facial plastic surgeon provided their clinical experience and consensus recommendations for applying *Aesthetic Treatment Considerations in the Perimenopause & Menopause Patient* into the aesthetic medical practice. The presenters were provided an opportunity to share knowledge while the viewing audience delivered questions. This activity was planned and implemented in accordance with the accreditation requirements and policies of the Accreditation Council for Continuing Medical Education (ACCME) through the joint providership of FabDay LLC and Advancing Knowledge in Healthcare, which is accredited by ACCME to provide continuing medical education for physicians.

## 3 Results/Discussion

### 3.1 Treatment Options

#### 3.1.1 Hormone Replacement Therapy (HRT)

Hormone replacement therapy is used to replace the estrogen the body stops making after menopause (Table 1). HRT is the most effective treatment for vasomotor symptoms and the genitourinary syndrome associated with menopause and can prevent bone loss and fractures [18]. Numerous forms of estrogen are available including conjugated equine estrogens, synthetic conjugated estrogens, micronized 17β‐estradiol, and ethinyl estradiol. Synthetic conjugated estrogens are comprised of estrone sulfate, equilin sulfate, and estradiol sulfate [18]. Progesterones are often added to prevent unopposed endometrial overgrowth and the increased risk of endometrial cancer during estrogen therapy. These include medroxyprogesterone acetate, norethindrone acetate, and micronized progesterone [18]. Bioidentical hormone therapy refers to BHRT often derived from plant sources like soy or yams, then processed in a lab to match the human hormone structure. BHRT used to treat menopausal symptoms may also be effective to some extent for improving menopausal skin symptoms (literature review) [19, 20].

**TABLE 1:** Summary of Therapeutic options for estrogen‐deficient skin: evidence levels and roundtable consensus recommendations.

Category	Treatments	Mechanism	Limitations	Evidence level
Hormonal therapy	Systemic estrogen, progesterone (under guidance of a gynecologist)	Replacing declining systemic estrogen to help mitigate collagen breakdown and improve vasomotor and cutaneous atrophy changes	Contraindicated in patients with history of thromboembolism, hormone‐sensitive cancer	Randomized clinical trials; literature reviews
Topical estrogen therapy	Estradiol; methyl estradiolpropanoate (MEP)	Directly activating cutaneous estrogen receptors to fibroblast and keratinocyte activation to improve skin collagen thickness and elasticity	Not FDA approved for anti‐aging; Limited safety data for systemic absorption	Randomized clinical trials; case series
Cosmeceuticals	Peptides, growth factors, exosomes	Stimulate fibroblasts and extra‐cellular remodeling	Variability among formulations; limited long‐term data	Split face clinical trials; comparative studies; literature reviews
Topical retinoids	Tretinoin, retinol, tazarotene	Binds nuclear retinoid receptors to help improve epidermal turnover, dermal collagen and elastin production	Irritation, erythema, peeling	Multiple randomized clinical trials, meta‐analysis
Biostimulators	Poly‐L‐lactic acid	Simulate fibroblast activity; provide extra‐cellular matrix scaffolding	May not be suitable for those with autoimmune disease history, risk of nodules	Randomized clinical trials; split‐body clinical trials
Injectable fillers	Hyaluronic acid	Provide hydration and may indirectly stimulate fibroblast activity	Delayed hypersensitivity, asymmetry, injection‐sites reactions; vascular occlusion	Retrospective cohort; case series
Energy‐based devices	CO_2_, radiofrequency, MFU‐V	Induce thermal remodeling, neocollagenesis to improve skin elasticity and texture	CO_2_ laser involves downtime as well as risk of prolonged erythema, infection, pigmentary changes	Systematic reviews; randomized clinical trials; expert consensus

Abbreviations: CO_2_, carbon dioxide laser; MFU‐V, microfocused ultrasound with visualization.

HRT has a storied history and had been controversial due to associations with adverse events including coronary heart disease, stroke, venous thromboembolism, and dementia [18]. These risks had prevented the use of oral HRT for dermatologic conditions [21]; however, more recent analyses indicate the absolute risks of adverse events following the initiation of HRT are much lower than previously thought [22]. HRT actually appears to be cardioprotective [23] and protective against dementia [24].

The risks of hormone therapy vary depending on type, dose, duration of use, route of administration, timing of initiation, and whether a progestogen is also used. HRT is currently recommended for treating troublesome vasomotor symptoms and prevention of bone loss in women < 60 years old or who are within 10 years of menopause onset [18]. Due to long‐term vasomotor symptoms (VMS), long‐duration hormone therapy use may be needed for some patients. Frequent VMS persist on average 7.4 years and more than 10 years for many patients [18].

Contraindications for oral and transdermal hormone therapy include unexplained vaginal bleeding, liver disease, prior estrogen‐sensitive cancer, coronary heart disease, stroke, myocardial infarction, or venous thromboembolism [18]. More information about HRT is available from the North American Menopause Society (www.Menopause.org).

#### 3.1.2 Topical Estrogen Therapy

The presence of two estrogen receptors (α and β) on dermal fibroblasts and epidermal keratinocytes [25] provides a rationale for the application of topical estrogen products for treating menopause‐related skin issues. Today, many pharmacies are compounding estrogen products prescribed for topical skin care. An extensive review of the literature revealed 23 clinical studies evaluating the effect of topical estrogen products on estrogen‐deficient skin (literature review) [26]. These included topical gels, ointments, patches, or creams which were applied most often to the face, but also arms, abdomen, buttocks, and hips. While studies may show them to be effective for reducing dryness and wrinkles [27], their off‐label use and variability in manufacturing protocols make them a less than ideal choice unless prescribed under the care of a knowledgeable and attentive expert. A consensus statement from the American College of Obstetricians and Gynecologists states “There is a lack of high‐quality data on the safety and efficacy of custom‐compounded bioidentical hormone therapy for the management of menopausal symptoms. Compounded bioidentical menopausal hormone therapy should not be prescribed routinely when FDA‐approved formulations exist” [28].

Some commercially available topical products contain methyl estradiolpropanoate, a cosmeceutical with estrogen‐like effects [29]. A double‐blind, placebo‐controlled clinical trial assessed the efficacy of topical methyl estradiolpropanoate in menopausal women 53–80 years old (randomized controlled trial) [30]. Following 14 weeks of twice‐daily facial application, subjects achieved significant improvement in baseline skin dryness, laxity, atrophy, and dullness versus placebo. Several subjects selected for a biopsy study demonstrated increased fibroblast estrogen receptor staining.

Compounds that bind to estrogen receptors and act as agonists or antagonists are called selective estrogen receptor modulators (SERMs) [31]. When they are derived from plants, they are referred to as phytoestrogens [32]. Isoflavones are phytoestrogens found in several plants in the legume family that act as SERMs. Numerous studies have shown the health benefits of orally administered isoflavones [33, 34]; however, they also have beneficial effects when applied topically [26]. The topical application of a proprietary product containing the phytoestrogen resveratrol has been assessed in moderately photodamaged skin (non‐randomized clinical trial) [35] After 12 weeks of daily application, subjects achieved a significant improvement in fine lines and wrinkles, skin firmness, skin elasticity, skin laxity, hyperpigmentation, radiance, and skin roughness. Genistein (5,7,4′‐trihydroxyisoflavone) is an isoflavone found in soybeans that functions as a SERM with a preference for estrogen receptor‐β [36]. Topical genistein may be useful for improving the quality, appearance, and health of the menopausal skin through its anti‐inflammatory and estrogen‐like effects (randomized clinical trial) [37].

#### 3.1.3 Topical Retinoids

Retinoids are considered the gold standard for dermatological conditions such as hyperpigmentation and photo‐aged skin [38]. Retinoids such as retinol can significantly increase epidermal thickness by stimulating keratinocytes' proliferation, reduce fine lines and wrinkles by activating dermal fibroblasts to produce type I collagen, fibronectin, and elastin, and normalize melanocyte function [39]. Commercially available topical retinoids for menopause include prescription tretinoin, tazarotene, and over‐the‐counter retinol and retinaldehyde (literature review) [40]. Unfortunately, retinoids can initially be quite irritating, and patients may experience facial redness, peeling, and dry skin for several weeks until the face becomes retinized. This may affect treatment compliance. As the potential for irritation is correlated with potency, retinol is better tolerated than retinoic acid [41].

#### 3.1.4 Topical Cosmeceuticals

Skin moisturizers can restore normal barrier function and decrease transepidermal water loss. Moisturizers typically contain occlusive ingredients such as dimethicone, shea butter, petrolatum, and humectants such as beta glucan, hyaluronic acid, and glycerin. As menopausal skin is more susceptible to reactive oxygen species and sun injury, moisturizers can be combined with antioxidants, anti‐inflammatory agents, and photoprotective sunscreens [42, 43, 44]. They can also serve as vehicles for other cosmeceutical products.

For example, cosmeceuticals can contain signal peptides which increase the production of glycosaminoglycans, collagen, elastin, and fibronectin. The most popular is palmitoyl pentapeptide (pam‐KTTKS), a procollagen I fragment that stimulates the production of collagen Types I, III, and IV. Used at a concentration of 4 PPM, it acts as a signal resulting in a cascading effect [45] to improve photoaged skin (randomized clinical trial) [46].

Cosmeceuticals can also be formulated with growth factors. The most commonly used growth factors are TGF‐β1 (transforming growth factor beta‐1) inducing keratinocyte migration, EGF (epidermal growth factor) inducing epidermal proliferation, PDGF (platelet‐derived growth factor) inducing macrophage activation and matrix production, and FGF (fibroblast growth factor) inducing fibroblast proliferation and migration (comparative study, literature review) [47, 48]. These products are safe and effective for facial rejuvenation [49, 50].

Finally, cosmeceuticals can be formulated to contain exosomes. Ranging in size from 30 to 150 nm, exosomes are membrane‐bound extracellular vesicles generated in the endosomal compartment of most eukaryotic cells [51]. Depending on their specific cell source, exosomes can contain many cell constituents including DNA, RNA, lipids, metabolites, and cell‐surface proteins. Currently, exosomes are derived primarily from bone marrow, placenta, adipose, and umbilical cord tissue [52]. Early preclinical studies using in vitro and animal models demonstrated the potential beneficial effects of exosomes on skin rejuvenation and hair growth (literature review) [53]. More recently, the safety and efficacy of a human platelet extract (HPE) was assessed for skin rejuvenation in an open‐label study including 48 women with a mean (SD) age of 54 (11) years (range, 40 to 80 years) (non‐randomized clinical trial) [54]. The study regimen was applied twice daily for 6 weeks. At that time, digital imaging revealed significant improvements in overall skin health which was correlated with significant reductions in skin redness, wrinkles, and melanin production and significant improvements in luminosity and color evenness. Despite the potential benefits of exosomes for aesthetic dermatology [55, 56], there have been no additional well‐controlled studies and there are currently no commercially available FDA‐approved products [57].

#### 3.1.5 Dermal Injectables

Injectables can also play a role in rejuvenating the premenopausal and menopausal face. Properly used dermal fillers can restore facial volume to create a natural, youthful appearance [58]. Depending on the type and method of administration, the benefit of fillers can range from correction of age‐related bone remodeling and muscle atrophy to treating fine lines and wrinkles (expert opinion, non‐randomized clinical trial) [59, 60].

Poly‐L‐lactic acid (PLLA‐SCA) is a regenerative injectable biostimulator used for reducing the severity of moderate to severe cheek lines and wrinkles [61, 62, 63] and restoring facial fat volume loss [64]. PLLA‐SCA stimulates neocollagenesis by causing a foreign body reaction to the injected polymer followed by a mild and controlled cellular inflammatory response leading to the formation of vascularized connective tissue [65]. Additionally, PLLA‐SCA stimulates adipogenesis and the expression of types IV and VI collagens [66]. A randomized, double‐blind study enrolled female subjects 30–60 years (*N* = 40) to receive facial PLLA‐SCA or saline injections every 4 weeks for 12 weeks (randomized clinical trial) [65]. At 12 months following their last injection, there were significant improvements in skin smoothness, elasticity, hydration in PLLA‐SCA‐treated subjects. Pigmentation, erythema, and pore size were significantly decreased. A recent randomized, double‐blinded split body trial evaluating PLLA‐SCA for treatment of hip dell volume deficit showed a significant improvement in dermal and adipose layer thickness with PLLA‐SCA compared to saline injection. This further supports PLLA‐SCA's impact on stimulating adipogenesis and elastogenesis (randomized clinical trial) [67]. For the changes commonly seen in menopausal skin, including decrease in dermal thickness and volume loss of the face, injectable PLLA‐SCA is a promising treatment option.

Intradermal hyaluronic acid (HA) filler injections have demonstrated the ability to improve skin texture, luminance, hydration, and elasticity [68]. An injectable crosslinked HA gel with lidocaine is intended for intradermal injection and designed to improve skin quality such as smoothness, hydration, and elasticity [69]. It incorporates short‐chain HA together with long‐chain HA to provide more efficient crosslinking than fillers based on other technologies. A retrospective study of subjects treated with this product to assess its aesthetic properties showed it to be useful for improving skin quality, requiring fewer and less frequent maintenance treatments. Subjects were primarily female (91.7%), with a mean (SD) age of 53.3 (11.8) years. No serious adverse events were reported (retrospective cohort study) [70].

Another technology has enabled the production of hybrid cooperative complexes of low and high molecular weight hyaluronans for injection into the facial fat compartments for adipose tissue restoration (retrospective case series) [71]. When administered to premenopausal and menopausal women with a mean age of 54 years (range, 40–70 years), subjects achieved significant improvements in Facial Volume Loss and Wrinkle Severity Rating Scales [72].

A new technique of fat grafting for the face called injectable tissue replacement and regeneration (ITR^2^) can specifically address the volume loss that occurs in different anatomic areas [73]. A 19‐month study assessed the short‐ and long‐term effects of facial fat grafting by ITR^2^ in the midfacial zone of female subjects (*N* = 29) with varying fat parcel sizes [74]. Subject data were analyzed collectively and by age (< 55 and > 55 years). There was an initial collective trend of volume loss during months 1–7 followed by a mean 52.3% increase during months 14 to 19 months. A similar trend was observed for subjects < 55 years of age, with a mean 54.1% post‐treatment gain and a final mean of 75.2%; however, subjects > 55 years of age experienced a linear decay from 60.6% to 29.5%.

#### 3.1.6 Energy‐Based Treatments

Finally, there is a broad range of energy‐based treatment options available for treating many of the menopause‐related skin issues such as skin laxity and hyperpigmentation [75]. These include multiple types of lasers [14, 76], radiofrequency devices [77, 78], microfocused ultrasound with visualization [15, 67], and intense pulsed light [79, 80].

Among these, CO_2_ laser resurfacing is considered the gold standard because of its ability to deliver deeper penetration, controlled ablation, and thermal injury. Ultrapulsed CO_2_ devices, in particular, ablate tissue quickly, provide excellent hemostasis, and deliver four times more energy per pulse than super‐pulsed lasers. This allows for more pronounced rejuvenation of areas such as periorbital skin, albeit with longer recovery and higher complication risk.

Another modality on the rise for treatment of menopausal skin, in particular targeting skin laxity, is microfocused ultrasound technology. Microfocused ultrasound with real‐time visualization (MFU‐V) delivers targeted thermal coagulation points at controlled depths within the dermis and subcutaneous tissue, inducing focal collagen denaturation [80]. This attracts fibroblasts, upregulates heat shock protein 47, and promotes both neocollagenesis and extracellular matrix remodeling. Recent histologic studies confirmed both new collagen formation and elastin neogenesis, with mature collagen and newly synthesized elastin fibers observed up to 90 days post‐treatment with MFU‐V, resulting in improved skin elasticity and lifting (systematic review) [80]. For this reason, MFU‐V is a viable regenerative treatment option for aging skin, with minimal associated downtime.

Lasers and energy‐based devices not only influence the physical structure of the skin, but also induce epigenetic modifications that stimulate cellular repair and regeneration [79]. By altering DNA methylation and histone modification patterns, these treatments can suppress genes associated with aging while activating those that promote collagen synthesis and extracellular matrix remodeling [79]. This epigenetic “reprogramming” of fibroblasts and keratinocytes contributes to long‐term improvements in skin structure and resilience.

## 4 Conclusion

Perimenopause and menopause are inevitable phases of every woman's life which are associated with numerous estrogen‐related changes including undesirable effects in the skin. This multidisciplinary roundtable, comprised of two dermatologists, a gynecologist, and a facial plastic surgeon, reviewed clinical experience and evidence to guide consensus recommendations for the aesthetic management of the skin during these life stages. The panel emphasized the importance of a multimodal approach combining both hormone‐based therapies along with procedural interventions (Table 1). Regenerative procedures included carbon dioxide resurfacing, poly‐L‐lactic acid injectables, and hyaluronic acid injectables. While no major disagreements emerged, this roundtable discussion emphasized the importance of hormonal balance on skin health. Hormone replacement therapy and topical estrogen directly address the hormonal deficiency, whereas regenerative procedures complement the hormones by offering tissue remodeling and collagen stimulation. Future research can prioritize randomized clinical trials in post‐menopausal women to better characterize efficacy in estrogen‐deficient skin. These changes, especially when affecting facial attractiveness, adversely affect the patient's quality of life. These combination approaches of systemic, topical, and procedural interventions can help mitigate the changes that can occur in estrogen‐deficient skin.

## Author Contributions

All authors contributed equally to the conception of the program, preparation of the manuscript, and approval of the version to be published.

## Disclosure

This activity was planned and implemented in accordance with the accreditation requirements and policies of the Accreditation Council for Continuing Medical Education (ACCME) through the joint providership of Advancing Knowledge in Healthcare (AKH Inc.) and FabDay LLC. AKH Inc. is accredited by the ACCME to provide continuing medical education for physicians.

## Ethics Statement

The authors confirm that the ethical policies of the journal, as noted on the journal's author guidelines page, have been adhered to. No ethical approval was required as this is a review article with no original research data.

## Consent

No informed consent was required as this is a review article with no original research data.

## Conflicts of Interest

Sabrina G. Fabi, MD, has received consulting fees from AbbVie Inc., Galderma, Merz Aesthetics, Revance Therapeutics Inc., Endo Pharmaceuticals, Croma Pharma, L'Oreal Groupe, Ortho Dermatologics, and RoC Skincare; is on the speaker's bureau for AbbVie Inc., Galderma, and Merz Aesthetics; and has received grants or research support from AbbVie Inc., Galderma, Merz Aesthetics, Symatese Aesthetics, Endo Pharmaceuticals, Croma Pharma, Teoxane, and Raziel Therapeutics; and is a stockholder in Allergan Aesthetics and Revance Therapeutics. Steven H. Dayan, MD, is a consultant to Allergan Aesthetics, Galderma, Merz Aesthetics, Evolus Inc., and Endo Pharmaceuticals; is on the speaker's bureau for Allergan Aesthetics, Galderma, and Merz Aesthetics; and has received grants and research support from Allergan Aesthetics, Galderma, Merz Aesthetics, Teoxane, Endo Pharmaceuticals, and Croma Pharma. Zoe D. Draelos, MD, is a researcher for Alloy Women's Health. Maya Firsowicz, MD, reports no financial relationships to disclose. Payvand Kamrani, DO, reports no financial relationships to disclose. The staff of Advancing Knowledge in Healthcare report no financial relationships to disclose.

## Data Availability

Data sharing not applicable to this article as no datasets were generated or analyzed during the current study.

## References

[1] M. Sussman , J. Trocio , C. Best , et al., “Prevalence of Menopausal Symptoms Among Mid‐Life Women: Findings From Electronic Medical Records,” BMC Women's Health 15 (2015): 58. 26271251 10.1186/s12905-015-0217-y PMC4542113

[2] C. M. C. de Santiago Nogueira , B. D. D. S. Silva , H. M. G. S. Costa , et al., “Clinical Profile and Quality of Life in Climacteric Women,” BMC Nursing 21 (2022): 233. 35999542 10.1186/s12912-022-00954-7 PMC9400204

[3] J. Whiteley , M. D. DiBonaventura , J. S. Wagner , J. Alvir , and S. Shah , “The Impact of Menopausal Symptoms on Quality of Life, Productivity, and Economic Outcomes,” Journal of Women's Health 22 (2013): 983–989. 10.1089/jwh.2012.3719 PMC3820128 24083674

[4] S. Li , K. Holm , M. Gulanick , and D. Lanuza , “Perimenopause and the Quality of Life,” Clinical Nursing Research 9 (2000): 6–23. 11271048 10.1177/105477380000900102

[5] J. L. Yang , E. Hodara , I. Sriprasert , D. Shoupe , and F. Z. Stanczyk , “Estrogen Deficiency in the Menopause and the Role of Hormone Therapy: Integrating the Findings of Basic Science Research With Clinical Trials,” Menopause 31 (2024): 926–939. 39081162 10.1097/GME.0000000000002407 PMC12072814

[6] F. Flament , R. Jiang , C. Delaunay , et al., “Evaluation of Adapted Dermocosmetic Regimens for Perimenopausal and Menopausal Women Using an Artificial Intelligence‐Based Algorithm and Quality of Life Questionnaires: An Open Observational Study,” Skin Research and Technology 29 (2023): e13349. 37522490 10.1111/srt.13349 PMC10293886

[7] S. Khosla , M. J. Oursler , and D. G. Monroe , “Estrogen and the Skeleton,” Trends in Endocrinology and Metabolism 23 (2012): 576–581. 22595550 10.1016/j.tem.2012.03.008 PMC3424385

[8] R. Malik , C. Pokeria , and S. Singh , “Correlation of Menopausal Symptoms With Serum Estradiol: A Study in Urban Indian Postmenopausal Women,” Journal of Obstetrics and Gynaecology of India 72 (2022): 322–329. 35923503 10.1007/s13224-021-01518-6 PMC9339428

[9] D. M. Reilly and J. Lozano , “Skin Collagen Through the Life Stages: Importance for Skin Health and Beauty,” Plastic and Aesthetic Research 8 (2021): 2.

[10] C. Castelo‐Branco , M. Duran , and J. González‐Merlo , “Skin Collagen Changes Related to Age and Hormone Replacement Therapy,” Maturitas 15 (1992): 113–119. 1345134 10.1016/0378-5122(92)90245-y

[11] M. J. Thornton , “Estrogens and Aging Skin,” Dermato‐Endocrinology 5 (2013): 264–270. 24194966 10.4161/derm.23872 PMC3772914

[12] E. D. Lephart , “A Review of the Role of Estrogen in Dermal Aging and Facial Attractiveness in Women,” Journal of Cosmetic Dermatology 17 (2018): 282–288. 29436770 10.1111/jocd.12508

[13] A. Scott and L. Newson , “Should We Be Prescribing Testosterone to Perimenopausal and Menopausal Women? A Guide to Prescribing Testosterone for Women in Primary Care,” British Journal of General Practice 70 (2020): 203–204. 10.3399/bjgp20X709265 PMC7098532 32217602

[14] R. M. Almukhtar , E. S. Wood , and S. G. Fabi , “Efficacy and Safety of Intense Pulsed Light and Nonablative Fractional 1440‐nm Diode Laser to a Combination of the 2 Modalities for Facial Rejuvenation,” Dermatologic Surgery 49 (2023): 42–47. 36533795 10.1097/DSS.0000000000003657

[15] S. G. Fabi , “Microfocused Ultrasound With Visualization for Skin Tightening and Lifting: My Experience and a Review of the Literature,” Dermatologic Surgery 40, no. 12 (2014): S164–S167. 25417569 10.1097/DSS.0000000000000233

[16] E. D. Lephart , “Bioactives for Estrogen‐Deficient Skin: Topical and Oral Supplement Clinical Studies. A Narrative Review,” Dermatologic Therapy 15 (2025): 1681–1703. 10.1007/s13555-025-01413-2 PMC12126418 40329055

[17] S. Fabi and S. Dayan , “Round Table Discussion: Aesthetic Considerations in the Perimenopause & Menopause Patient. June 13, 2025,” (2025), https://events.avenue.live/.

[18] Advisory Panel of the North American Menopause Society , “The 2022 Hormone Therapy Position Statement of the North American Menopause Society,” Menopause 29 (2022): 767–794. 35797481 10.1097/GME.0000000000002028

[19] M. P. Brincat , Y. M. Baron , and R. Galea , “Estrogens and the Skin,” Climacteric 8 (2005): 110–123. 16096167 10.1080/13697130500118100

[20] E. K. Merzel Šabović , T. Kocjan , and I. Zalaudek , “Treatment of Menopausal Skin—A Narrative Review of Existing Treatments, Controversies, and Future Perspectives,” Post Reproductive Health 30 (2024): 85–94. 38379168 10.1177/20533691241233440

[21] G. V. Duarte , A. C. Trigo , and M. D. F. P. de Oliveira , “Skin Disorders During Menopause,” Cutis 97 (2016): E16–E23. 26919507

[22] L. Cho , A. M. Kaunitz , S. S. Faubion , et al., “Rethinking Menopausal Hormone Therapy: For Whom, What, When, and How Long?,” Circulation 147 (2023): 597–610. 36780393 10.1161/CIRCULATIONAHA.122.061559 PMC10708894

[23] H. N. Hodis and W. J. Mack , “Menopausal Hormone Replacement Therapy and Reduction of All‐Cause Mortality and Cardiovascular Disease: It Is About Time and Timing,” Cancer Journal 28 (2022): 208–223. 35594469 10.1097/PPO.0000000000000591 PMC9178928

[24] N. Ali , R. Sohail , S. R. Jaffer , et al., “The Role of Estrogen Therapy as a Protective Factor for Alzheimer's Disease and Dementia in Postmenopausal Women: A Comprehensive Review of the Literature,” Cureus 15 (2023): e43053. 37680393 10.7759/cureus.43053 PMC10480684

[25] L. R. Nelson and S. E. Bulun , “Estrogen Production and Action,” Journal of the American Academy of Dermatology 45, no. 3 (2001): S116–S124. 11511861 10.1067/mjd.2001.117432

[26] A. K. Rzepecki , J. E. Murase , R. Juran , S. G. Fabi , and B. N. McLellan , “Estrogen‐Deficient Skin: The Role of Topical Therapy,” International Journal of Women's Dermatology 5 (2019): 85–90. 10.1016/j.ijwd.2019.01.001 PMC6451761 30997378

[27] P. Creidi , B. Faivre , P. Agache , E. Richard , V. Haudiquet , and J. P. Sauvanet , “Effect of a Conjugated Oestrogen (Premarin) Cream on Ageing Facial Skin. A Comparative Study With a Placebo Cream,” Maturitas 19 (1994): 211–223. 7799828 10.1016/0378-5122(94)90074-4

[28] Committee on Clinical Consensus–Gynecology , “Compounded Bioidentical Menopausal Hormone Therapy: ACOG Clinical Consensus No. 6,” Obstetrics and Gynecology 142 (2023): 1266–1273. 37856860 10.1097/AOG.0000000000005395

[29] J. Cohen and J. Downie , “An Open‐Label Study Evaluating the Periorbital Skin Rejuvenation Efficacy of a Cosmeceutical Containing Methyl Estradiolpropanoate (MEP) in Women With Estrogen Deficient Skin (EDS),” Journal of Drugs in Dermatology 21 (2022): 1185–1190. 36342739 10.36849/JDD.7279

[30] Z. D. Draelos , “A Double‐Blind Randomized Pilot Study Evaluating the Safety and Efficacy of Topical MEP in the Facial Appearance Improvement of Estrogen Deficient Females,” Journal of Drugs in Dermatology 17 (2018): 1186–1189. 30500138

[31] E. D. Lephart and F. Naftolin , “Factors Influencing Skin Aging and the Important Role of Estrogens and Selective Estrogen Receptor Modulators (SERMs),” Clinical, Cosmetic and Investigational Dermatology 15 (2022): 1695–1709. 36017417 10.2147/CCID.S333663 PMC9397534

[32] T. Oseni , R. Patel , J. Pyle , and V. C. Jordan , “Selective Estrogen Receptor Modulators and Phytoestrogens,” Planta Medica 74 (2008): 1656–1665. 18843590 10.1055/s-0028-1088304 PMC2587438

[33] M. Wójciak , P. Drozdowski , A. Skalska‐Kamińska , et al., “Protective, Anti‐Inflammatory, and Anti‐Aging Effects of Soy Isoflavones on Skin Cells: An Overview of In Vitro and In Vivo Studies,” Molecules 29 (2024): 5790. 39683947 10.3390/molecules29235790 PMC11643064

[34] J. Rizzo , M. Min , S. Adnan , et al., “Soy Protein Containing Isoflavones Improves Facial Signs of Photoaging and Skin Hydration in Postmenopausal Women: Results of a Prospective Randomized Double‐Blind Controlled Trial,” Nutrients 15 (2023): 4113. 37836398 10.3390/nu15194113 PMC10574417

[35] P. Farris , M. Yatskayer , N. Chen , Y. Krol , and C. Oresajo , “Evaluation of Efficacy and Tolerance of a Nighttime Topical Antioxidant Containing Resveratrol, Baicalin, and Vitamin e for Treatment of Mild to Moderately Photodamaged Skin,” Journal of Drugs in Dermatology 13 (2014): 1467–1472. 25607790

[36] M. S. Nestor , V. Bhupalam , N. Awad , and J. D. Hetzel , “The Therapeutic Role of Genistein in Perimenopausal and Postmenopausal Women,” Journal of Clinical and Aesthetic Dermatology 17 (2024): 45–53. PMC11495164 39445324

[37] M. Na Takuathung , P. Klinjan , W. Sakuludomkan , et al., “Efficacy and Safety of the Genistein Nutraceutical Product Containing Vitamin E, Vitamin B3, and Ceramide on Skin Health in Postmenopausal Women: A Randomized, Double‐Blind, Placebo‐Controlled Clinical Trial,” Journal of Clinical Medicine 12 (2023): 1326. 36835861 10.3390/jcm12041326 PMC9963595

[38] T. Quan , “Human Skin Aging and the Anti‐Aging Properties of Retinol,” Biomolecules 13 (2023): 1614. 38002296 10.3390/biom13111614 PMC10669284

[39] Y. Shao , T. He , G. J. Fisher , J. J. Voorhees , and T. Quan , “Molecular Basis of Retinol Anti‐Ageing Properties in Naturally Aged Human Skin In Vivo,” International Journal of Cosmetic Science 39 (2017): 56–65. 27261203 10.1111/ics.12348 PMC5136519

[40] S. Mukherjee , A. Date , V. Patravale , H. C. Korting , A. Roeder , and G. Weindl , “Retinoids in the Treatment of Skin Aging: An Overview of Clinical Efficacy and Safety,” Clinical Interventions in Aging 1 (2006): 327–348. 18046911 10.2147/ciia.2006.1.4.327 PMC2699641

[41] R. R. Riahi , A. E. Bush , and P. R. Cohen , “Topical Retinoids: Therapeutic Mechanisms in the Treatment of Photodamaged Skin,” American Journal of Clinical Dermatology 17 (2016): 265–276. 26969582 10.1007/s40257-016-0185-5

[42] F. A. S. Addor , “Antioxidants in Dermatology,” Anais Brasileiros de Dermatologia 92 (2017): 356–362. 29186248 10.1590/abd1806-4841.20175697 PMC5514576

[43] N. Chandan , J. R. Rajkumar , V. Y. Shi , and P. A. Lio , “A New Era of Moisturizers,” Journal of Cosmetic Dermatology 20 (2021): 2425–2430. 33977643 10.1111/jocd.14217

[44] S. G. Fabi , L. Cattelan , B. Caughlin , J. S. Pérez , M. Cirrincione , and S. Dayan , “Unlocking the Psycho‐Social‐Dermal Axis: A Double Blinded Randomized Placebo Controlled Study Unveiling the Influence of a Novel Topical Formulation on Skin Quality, Attractiveness, Quality of Life, and Sexual Satisfaction,” Journal of Cosmetic Dermatology 23 (2024): 2905–2917. 39073288 10.1111/jocd.16496

[45] K. Katayama , J. Armendariz‐Borunda , R. Raghow , A. H. Kang , and J. M. Seyer , “A Pentapeptide From Type I Procollagen Promotes Extracellular Matrix Production,” Journal of Biological Chemistry 268 (1993): 9941–9944. 8486721

[46] L. R. Robinson , N. C. Fitzgerald , D. G. Doughty , N. C. Dawes , C. A. Berge , and D. L. Bissett , “Topical Palmitoyl Pentapeptide Provides Improvement in Photoaged Human Facial Skin,” International Journal of Cosmetic Science 27 (2005): 155–160. 18492182 10.1111/j.1467-2494.2005.00261.x

[47] M. S. Kim , H. J. Song , S. H. Lee , and C. K. Lee , “Comparative Study of Various Growth Factors and Cytokines on Type I Collagen and Hyaluronan Production in Human Dermal Fibroblasts,” Journal of Cosmetic Dermatology 13 (2014): 44–51. 24641605 10.1111/jocd.12073

[48] O. Eskens and S. Amin , “Challenges and Effective Routes for Formulating and Delivery of Epidermal Growth Factors in Skin Care,” International Journal of Cosmetic Science 43 (2021): 123–130. 33354795 10.1111/ics.12685

[49] D. J. Quinlan , A. M. Ghanem , and H. Hassan , “Topical Growth Factor Preparations for Facial Skin Rejuvenation: A Systematic Review,” Journal of Cosmetic Dermatology 22 (2023): 2023–2039. 37222303 10.1111/jocd.15644

[50] D. C. Wu and M. P. Goldman , “A Prospective, Randomized, Double‐Blind, Split‐Face Clinical Trial Comparing the Efficacy of Two Topical Human Growth Factors for the Rejuvenation of the Aging Face,” Journal of Clinical and Aesthetic Dermatology 10 (2017): 31–35. PMC5479475 28670356

[51] M. Yáñez‐Mó , P. R. Siljander , Z. Andreu , et al., “Biological Properties of Extracellular Vesicles and Their Physiological Functions,” Journal of Extracellular Vesicles 4 (2015): 27066. 25979354 10.3402/jev.v4.27066 PMC4433489

[52] Y. C. Ku , H. Omer Sulaiman , S. R. Anderson , and A. R. Abtahi , “The Potential Role of Exosomes in Aesthetic Plastic Surgery: A Review of Current Literature,” Plastic and Reconstructive Surgery. Global Open 11 (2023): e5051. 37313480 10.1097/GOX.0000000000005051 PMC10259637

[53] N. Hartman , J. Loyal , and S. Fabi , “Update on Exosomes in Aesthetics,” Dermatologic Surgery 48 (2022): 862–865. 35580250 10.1097/DSS.0000000000003487

[54] S. L. Proffer , C. R. Paradise , E. DeGrazia , et al., “Efficacy and Tolerability of Topical Platelet Exosomes for Skin Rejuvenation: Six‐Week Results,” Aesthetic Surgery Journal 42 (2022): 1185–1193. 35689936 10.1093/asj/sjac149

[55] R. H. Mahmoud , E. Peterson , E. V. Badiavas , M. Kaminer , and A. E. Eber , “Exosomes: A Comprehensive Review for the Practicing Dermatologist,” Journal of Clinical and Aesthetic Dermatology 18 (2025): 33–40. PMC12007658 40256340

[56] M. Shah , V. Dukharan , L. Broughton , et al., “Exosomes for Aesthetic Dermatology: A Comprehensive Literature Review and Update,” Journal of Cosmetic Dermatology 24 (2025): e16766. 39764639 10.1111/jocd.16766 PMC11704993

[57] Food and Drug Administration , “Consumer Alert on Regenerative Medicine Products Including Stem Cells and Exosomes, April 09, 2024,” (2025), https://www.fda.gov/vaccines‐blood‐biologics/consumers‐biologics/consumer‐alert‐regenerative‐medicine‐products‐including‐stem‐cells‐and‐exosomes.

[58] C. Muhn , N. Rosen , N. Solish , et al., “The Evolving Role of Hyaluronic Acid Fillers for Facial Volume Restoration and Contouring: A Canadian Overview,” Clinical, Cosmetic and Investigational Dermatology 5 (2012): 147–158. 23071398 10.2147/CCID.S30794 PMC3469309

[59] K. Goldie , W. Peeters , M. Alghoul , et al., “Global Consensus Guidelines for the Injection of Diluted and Hyperdiluted Calcium Hydroxylapatite for Skin Tightening,” Dermatologic Surgery 44, no. 1 (2018): S32–S41. 30358631 10.1097/DSS.0000000000001685

[60] D. S. Müller , V. Prinz , M. Sulovsky , M. Cajkovsky , S. Cotofana , and K. Frank , “Volumization of the Young and the Old Temple Using a Highly Cross‐Linked HA Filler,” Journal of Cosmetic Dermatology 20 (2021): 1634–1642. 33773034 10.1111/jocd.14109

[61] S. Fabi , T. Hamilton , B. LaTowsky , et al., “Effectiveness and Safety of Sculptra Poly‐L‐Lactic Acid Injectable Implant in the Correction of Cheek Wrinkles,” Journal of Drugs in Dermatology 23 (2024): 1297–1305. 38206151 10.36849/JDD.7729

[62] J. Waibel , M. Ziegler , T. Q. Nguyen , et al., “Comparative Bulk RNA‐Seq Analysis of Poly‐l‐Lactic Acid Versus Calcium Hydroxylapatite Reveals a Novel, Adipocyte‐Mediated Regenerative Mechanism of Action Unique to PLLA,” Dermatologic Surgery 50 (2024): S166–S171. 39480040 10.1097/DSS.0000000000004425

[63] J. Waibel , T. Q. Nguyen , J. H. T. D. Le , et al., “Gene Analysis of Biostimulators: Poly‐L‐Lactic Acid Triggers Regeneration While Calcium Hydroxylapatite Induces Inflammation Upon Facial Injection,” Journal of Drugs in Dermatology 24 (2025): 34–40. 39761144 10.36849/JDD.8464

[64] R. Zubair , L. Ishii , J. Loyal , N. Hartman , and S. G. Fabi , “SPLASH: Split‐Body Randomized Clinical Trial of Poly‐l‐Lactic Acid for Adipogenesis and Volumization of the Hip Dell,” Dermatologic Surgery 50 (2024): 1155–1162. 39503574 10.1097/DSS.0000000000004417

[65] K. Bohnert , A. Dorizas , P. Lorenc , and N. S. Sadick , “Randomized, Controlled, Multicentered, Double‐Blind Investigation of Injectable Poly‐L‐Lactic Acid for Improving Skin Quality,” Dermatologic Surgery 45 (2019): 718–724. 30741790 10.1097/DSS.0000000000001772

[66] H. W. Kim , Y. A. Jung , J. M. Yun , et al., “Effects of Poly‐L‐Lactic Acid on Adipogenesis and Collagen Gene Expression in Cultured Adipocytes Irradiated With Ultraviolet B Rays,” Annals of Dermatology 35 (2023): 424–431. 38086356 10.5021/ad.22.177 PMC10733076

[67] S. G. Fabi , “Noninvasive Skin Tightening: Focus on New Ultrasound Techniques,” Clinical, Cosmetic and Investigational Dermatology 8 (2015): 47–52. 25709486 10.2147/CCID.S69118 PMC4327394

[68] L. Nakab , C. K. Hee , and O. Guetta , “Improvements in Skin Quality Biological Markers in Skin Explants Using Hyaluronic Acid Fller VYC‐12L,” Plastic and Reconstructive Surgery ‐ Global Open 8 (2020): e2723. 32537370 10.1097/GOX.0000000000002723 PMC7253252

[69] F. Niforos , P. Ogilvie , M. Cavallini , et al., “VYC‐12 Injectable Gel Is Safe and Effective for Improvement of Facial Skin Topography: A Prospective Study,” Clinical, Cosmetic and Investigational Dermatology 12 (2019): 791–798. 31749628 10.2147/CCID.S216222 PMC6817835

[70] D. Dal'Asta Coimbra , “Clinical Experience With a New Injectable Hyaluronic Acid Designed to Improve Skin Quality in a Private Clinic in Brazil: A Retrospective Cohort Study,” Health Science Reports 4 (2021): e399. 34632098 10.1002/hsr2.399 PMC8493242

[71] D. Cassuto , C. Cigni , G. Bellia , and C. Schiraldi , “Restoring Adipose Tissue Homeostasis in Response to Aging: Initial Clinical Experience With Profhilo Structura,” Gels 9 (2023): 614. 37623069 10.3390/gels9080614 PMC10453823

[72] A. Sparavigna , F. Grimolizzi , C. Cigni , R. Lualdi , and G. Bellia , “Efficacy and Tolerability of Profhilo Structura Intended to Restore Lateral Cheek Fat Compartment: An Observational Pilot Study,” Health Science Reports 7 (2024): e1743. 38260185 10.1002/hsr2.1743 PMC10801275

[73] J. S. Crowley , E. Kream , S. Fabi , and S. R. Cohen , “Facial Rejuvenation With Fat Grafting and Fillers,” Aesthetic Surgery Journal 41, no. 1 (2021): S31–S38. 34002771 10.1093/asj/sjab014

[74] S. R. Cohen , J. Wesson , S. Willens , et al., “Standardized Anatomic and Regenerative Facial Fat Grafting: Objective Photometric Evaluation 1 to 19 Months After Injectable Tissue Replacement and Regeneration,” Aesthetic Surgery Journal 42 (2022): 327–339. 34724035 10.1093/asj/sjab379

[75] Y. S. Levin , “Cosmetic Dermatology in Menopause,” Menopause 29 (2022): 344–350. 35013059 10.1097/GME.0000000000001925

[76] T. L. I. Levy , J. Waibel , G. G. Gauglitz , et al., “Expert Consensus on Clinical Recommendations for Fractional Ablative CO_2_ Laser in Facial Skin Rejuvenation Treatment,” Lasers in Surgery and Medicine 57 (2025): 15–26. 39434507 10.1002/lsm.23850 PMC11776446

[77] J. Carruthers , S. Fabi , and R. Weiss , “Monopolar Radiofrequency for Skin Tightening: Our Experience and a Review of the Literature,” Dermatologic Surgery 40, no. 12 (2014): S168–S173. 25417570 10.1097/DSS.0000000000000232

[78] J. Dendle , D. C. Wu , S. G. Fabi , D. Melo , and M. P. Goldman , “A Retrospective Evaluation of Subsurface Monopolar Radiofrequency for Lifting of the Face, Neck, and Jawline,” Dermatologic Surgery 42 (2016): 1261–1265. 27598446 10.1097/DSS.0000000000000858

[79] L. Zaleski , S. Fabi , and M. P. Goldman , “Treatment of Melasma and the Use of Intense Pulsed Light: A Review,” Journal of Drugs in Dermatology 11 (2012): 1316–1320. 23135081

[80] A. F. S. Sales , I. L. Pandolfo , M. de Almeida Cruz , et al., “Intense Pulsed Light on Skin Rejuvenation: A Systematic Review,” Archives of Dermatological Research 314 (2022): 823–838. 34609598 10.1007/s00403-021-02283-2

